# Linking unfounded beliefs to genetic dopamine availability

**DOI:** 10.3389/fnhum.2015.00521

**Published:** 2015-09-30

**Authors:** Katharina Schmack, Hannes Rössler, Maria Sekutowicz, Eva J. Brandl, Daniel J. Müller, Predrag Petrovic, Philipp Sterzer

**Affiliations:** ^1^Department of Psychiatry, Charité Campus Mitte, Charité Universitätsmedizin BerlinBerlin, Germany; ^2^Neurogenetics Section, Centre for Addiction and Mental HealthToronto, ON, Canada; ^3^Department of Clinical Neuroscience, Karolinska InstitutetStockholm, Sweden

**Keywords:** dopamine, COMT, haplotype, visual perception, beliefs

## Abstract

Unfounded convictions involving beliefs in the paranormal, grandiosity ideas or suspicious thoughts are endorsed at varying degrees among the general population. Here, we investigated the neurobiopsychological basis of the observed inter-individual variability in the propensity toward unfounded beliefs. One hundred two healthy individuals were genotyped for four polymorphisms in the COMT gene (*rs6269, rs4633, rs4818*, and *rs4680*, also known as *val*^158^*met*) that define common functional haplotypes with substantial impact on synaptic dopamine degradation, completed a questionnaire measuring unfounded beliefs, and took part in a behavioral experiment assessing perceptual inference. We found that greater dopamine availability was associated with a stronger propensity toward unfounded beliefs, and that this effect was statistically mediated by an enhanced influence of expectations on perceptual inference. Our results indicate that genetic differences in dopaminergic neurotransmission account for inter-individual differences in perceptual inference linked to the formation and maintenance of unfounded beliefs. Thus, dopamine might be critically involved in the processes underlying one's interpretation of the relationship between the self and the world.

## Introduction

Beliefs constitute a representation of the world and provide the basis for the experience to be a self within it (Kircher and Leube, 2003; Connors and Halligan, 2014). However, although confronted with the same external reality, humans differ in their beliefs about the world. For instance, while many people passionately believe in spiritual healing, horoscopes or telepathy, others vehemently reject the idea of phenomena that are not directly supported by empirical evidence. Unfounded convictions involving beliefs in the paranormal, grandiosity ideas or suspicious thoughts are endorsed at varying degrees by individuals of the general population (Peters et al., 1999). Interestingly, there is converging evidence that such unfounded beliefs in the healthy lie on a continuum with delusions in individuals suffering from psychosis (Freeman, 2006; Linscott and van Os, 2013; Zavos et al., 2014), and alterations in the self-related processes have been demonstrated for both unfounded beliefs in the healthy (Fenigstein and Vanable, 1992) and delusions in psychotic individuals (Smári et al., 1994). This suggests that the tendency toward unfounded beliefs represents a continuously distributed phenotype that in its extreme form can lead to significant distress and social isolation. However, the neurobiological bases underlying the observed inter-individual variability in the propensity toward unfounded beliefs have remained elusive.

Influential theories of brain function describe beliefs as an internal model of the world that serves to generate expectations that structure incumbent sensory information in order to create meaningful perceptual experiences (Mumford, 1992; Friston, 2005). Accordingly, a stronger influence of expectations on sensory processing would result in the tendency to perceive the world to a greater extent in conformity with expected meaning and to a lesser extent in conformity with the external reality, thus leading to unfounded beliefs. In support of this theory, empirical work has consistently linked unfounded beliefs with an increased influence of expectations on visual perception. For instance, paranormal beliefs and magical ideation have been associated to an increased willingness to perceive expected yet non-existent contents in visual noise (Tsakanikos and Reed, 2005; Krummenacher et al., 2010; Riekki and Lindeman, 2013; Van Elk, 2013). Furthermore, we have recently shown that the tendency toward delusional ideation in healthy individuals is related to the effect of experimentally induced cognitive expectations on reported perception, and, notably, also on early visual processing (Schmack et al., 2013a). This suggests that individual differences in the propensity toward unfounded beliefs are related to individual differences in the neural mechanisms that govern visual perception.

**Figure 1:**
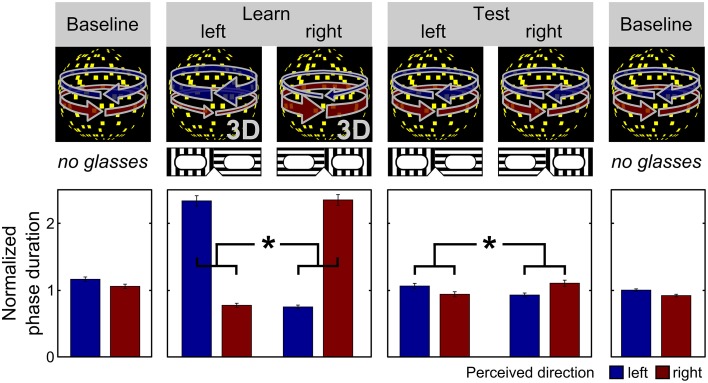
**Schematic illustration of the behavioral experiment**. Expectations about the appearance of the visual stimulus were induced by a placebo-like paradigm (as similarly described in Schmack et al., 2013a). Participants viewed continuously a dot-kinematogramm that is perceived as a rotating sphere and indicated changes in perceived rotation direction by key presses. In the initial and final baseline phase, the rotation direction of the sphere was ambiguous yielding bistable perception alternating spontaneously between leftward and rightward rotation direction. On average, both rotation directions were perceived for equally long phase durations in the baseline phases. In the learning phase, participants wore transparent glasses which they believed to contain polarizing filters and bias their perception toward one rotation direction depending on the orientation of the glasses. Stereoscopic depth cues were surreptitiously added to the stimulus, which forced the sphere to rotate into one direction for 80% of the time resulting in longer average phase durations of the expected rotation direction compared to the unexpected rotation direction. Critically, in the test phase, participants again wore the transparent glasses that they expected to bias their perception but were now again shown with the sphere with ambiguous rotation direction. Again, participants perceived the expected rotation direction for longer average phase durations than the unexpected rotation direction, which in the absence of any depth cues in the stimulus can only be attributed to the participants' expectations associated with the glasses (^*^p < 0.001, paired *t*-test, *p*-value based on 10,000 permutations; the bars show the mean phase duration of each percept normalized with respect to the mean phase duration in the baseline runs; the error bars denote SEM).

Here, we sought to elucidate the neurobiological underpinnings of the link between perceptual inference mechanisms and unfounded beliefs. As dopamine (DA) has consistently been implicated in the formation of pathological forms of unfounded beliefs, i.e., delusions (for recent reviews see Howes and Kapur, 2009; Pankow et al., 2012), we asked whether genetic differences in dopaminergic neurotransmission might provide the basis for differences in perceptual inference and thereby explain the observed inter-individual variability in the tendency toward unfounded beliefs. One hundred two healthy individuals were genotyped for common functional haplotypes of catechol-O-methyltransferase (COMT) that account for significant variations in DA degradation (Diatchenko et al., 2005; Nackley et al., 2006), completed a validated questionnaire that measures the tendency toward unfounded, delusion-like beliefs (Peters et al., 1999), and took part in a visual perception experiment that assessed the role of cognitive expectations in perceptual inference (Figure 1, Sterzer et al., 2008; Schmack et al., 2013a). We hypothesized that greater genetically determined DA availability would be associated with a stronger tendency toward unfounded beliefs, and that this association would be mediated by a stronger effect of cognitive expectations in perceptual inference.

## Materials and methods

### Participants

One hundred nine healthy volunteers were recruited via mailing lists of Charité - Universitätsmedizin Berlin and Humboldt-Universität zu Berlin. Seven participants were excluded from further analyses due to technical problems during data collection or haplotype analysis (see below) yielding a final sample of 102 participants (53 females, 24.9 ± 4.7 years [mean ± SD]). Behavioral results from the same sample, i.e., the effect of learned expectations on perception and its correlation with the tendency toward unfounded beliefs (see Section COMT Haplotype Distribution), but not the genetic data were presented in a previous publication (Schmack et al., 2013a). The current sample (*n* = 102) lacked 4 participants of the previous sample that were excluded due to problems during genotyping (see Section Participants). In addition, the current sample (*n* = 102) included one participant that was not included in the previous sample due to problems in data collection during an additional experiment presented in our previous work but not here (see Section “Materials and Methods, Experimental design, Behavioral Experiment 1” in Schmack et al., 2013a). All participants were naive with regard to the purpose of the experiment, had normal or corrected-to-normal vision, and no Axis I psychiatric disorder (Structured Clinical Interview for Diagnostic and Statistical Manual of Mental Disorders, Fourth Edition, Axis I Disorders). Participants gave written informed consent and received monetary remuneration for their time. The study was approved by the Ethics Committee of Charité - Universitätsmedizin Berlin and conducted in accordance with the declaration of Helsinki.

### Genotype and haplotype estimation

To address genetic differences in DA neurotransmission, we analyzed four single nucleotide polymorphisms (SNPs) of the DA degrading enzyme COMT that form previously described haplotypes with functional consequences on enzymatic protein function (Diatchenko et al., 2005). A fifth frequent SNP in the COMT gene was analyzed for control purposes. DNA was extracted from venous blood samples following a standard high-salt procedure. Genotyping was conducted using TaqMan® assays and was executed on an ABI Prism® 7500 Sequence Detection System with an Allelic Discrimination program within the ABI software (Applied Biosystems, Foster City, CA). Four SNPs defining the previously described haplotype were genotyped: rs6269 (−98A>G), rs4633 (186C>T, his62his), rs4818 (408C>G, leu136 leu), rs4860 (472G>A, val158met). To test whether we could reproduce the previously defined haplotypes (Diatchenko et al., 2005) in our sample, we genotyped an additional control SNP in the COMT gene (rs165599, 522G>A) which we expected not to form part of the haploblock. None of the SNPs deviated significantly from Hardy-Weinberg equilibrium (*p* > 0.1, chi-square test). Haplotype block analysis was performed using the software Haploview 4.2 (Barrett et al., 2005) and revealed three common haplotypes in line with previous work (Diatchenko et al., 2005):

1. high enzymatic activity haplotype (also known as ‘low pain sensitivity’ [LPS]): *rs6269/G-rs4633/C-rs4818/G-rs4680/G*
2. intermediate enzymatic activity haplotype (also known as ‘average pain sensitivity’ = APS): *rs6269/A-rs4633/T-rs4818/C-rs4680/A*
3. low enzymatic activity haplotype (also known as ‘high pain sensitivity’ = HPS): *rs6269/A-rs4633/C-rs4818/C- rs4680/G*

Haplotypes were reconstructed for each participant using the software PHASE v2.1 (Stephens and Donnelly, 2003). Four participants with ambiguous haplotypes as indicated by confidence values < 90% were excluded from further analysis.

### Measurement of unfounded beliefs

To quantify each participant's tendency toward unfounded beliefs, we administered the Peters Delusions Inventory (PDI) (Peters et al., 1999). The 40 items of this self-rating questionnaire cover a wide range of common unfounded beliefs with delusion-like contents, including beliefs in the paranormal, grandiosity ideas or suspicious thoughts. Items are prefaced with a relative (“*Do you ever feel as if…?*”) which ensures the measurement of the propensity toward unfounded beliefs rather than of the present endorsement of such beliefs. In addition to simple yes-no statements, the questionnaire asks for dimensional ratings assessing the degree of distress, preoccupation and conviction associated with each endorsed belief. As a previous analysis of the same data set had shown that conviction scores were most tightly linked to inter-individual differences in perceptual inference (Schmack et al., 2013a), only conviction scores were included into statistical analysis.

### Behavioral experiment

Participants took part in a behavioral experiment in which we induced expectations about a visual ambiguous stimulus using a placebo-like manipulation (Sterzer et al., 2008; Schmack et al., 2013a). In brief, expectations were induced by presenting stimuli that were truly biased in terms of their visual motion. However, the subjects were told that this bias was caused by glasses through which they viewed the stimuli. Crucially, in a subsequent session, although the subjects retained their glasses (and expectations about the prevalent direction of motion), the stimuli actually presented were not biased. This enabled us to assess the impact of expectations induced by prior experience on perception of an unbiased stimulus.

As similarly, described in a previous study with a slightly extended sample (Schmack et al., 2013a), visual stimuli were presented using MATLAB (MathWorks Inc.) and the Cogent 2000 toolbox (http://www.vislab.ucl.ac.uk/cogent.php) in a room with a constant low-light situation on a Samsung ® CRT display (refresh rate 60 Hz, resolution 1024 × 768 pixels). Stimuli were shown dichoptically through a mirror stereoscope allowing to control the visual input to each eye separately. Stimuli were dot-kinematograms (DKs) that were orthographic projections of a sphere rotating around a vertical axis (diameter 4.1° of visual angle, rotation speed 1/6 revolutions/s) and consisted of 450 randomly distributed yellow square “dots” (total size 4.1° maximum 0.2° × 0.2°) moving coherently left- or rightward on a black background with a central fixation cross and framed by white square.

Depending on the experimental run (see below), rotation direction of the sphere could be either ambiguous or unambiguous. The ambiguous sphere rotating around the vertical axis consisted of two identical DKs presented to each eye. The ambiguous sphere yields bistable perception alternating spontaneously between the two rotation directions. To produce the unambiguous sphere used for the experimental induction of expectations in the learning runs of the experiment (see below), two slightly different DKs were displayed representing two different perspectives (maximal offset 0.5°). This interocular disparity minimizes the ambiguity of rotation direction. To mimic the spontaneous perceptual alternations that occur during viewing of the ambiguous sphere, the unambiguous sphere alternated between both rotation directions with an overall rate comparable to the subject's switch rate but with different dominance times (80% vs. 20% on average). Debriefing after the experiment indicated that the majority (80 out of 102) of the participants did not notice any difference between the ambiguous and the unambiguous sphere. Of those who noticed a difference, the majority (16 out of 22) attributed it to subjective factors such as habituation or fatigue.

Before the experiment, participants were informed that they would perform an experiment on depth perception. They were told that they would view a bistable rotating sphere and were instructed to maintain fixation and indicate changes in perceived rotation direction by key presses. They were told that standard 3D-glasses would be used in some runs of the experiment. These glasses, which were in reality completely transparent, would contain two different filters so that one eye would be only reached by horizontally polarized light and the other eye only by vertically polarized light. Due to stereoscopic vision induced by these glasses their perception would be biased toward one rotation direction. Which rotation direction this was would depend on which eye looked through which filter, and orientation of the glasses would be manually reversed during the experiment.

During each experimental run, the stimulus was presented continuously for 240 s. The whole experiment consisted of three initial baseline runs, during which the ambiguous DK was presented, followed by two unambiguous learning runs, two ambiguous test runs, and two final ambiguous baseline runs (Figure 1). In the learning and test runs subjects wore the transparent glasses which they believed to contain polarizing filters. The actual (learning phase) or expected (test phase) dominant rotation directions in each run were contingent on the orientation of the transparent glasses and were switched between the runs resulting in one learning run and two test runs per orientation, respectively. The association between orientations and rotation directions was counterbalanced across participants to control for systematic effects of the glasses on perception. The order of dominant directions across runs was pseudo-randomized and balanced across participants to preclude systematic effects of the dominant direction in the second learning run on the following test runs. Participants indicated perceptual transitions by key presses choosing between three response options: leftward rotation, rightward rotation and uncertain perceptual state.

As a measure of the effect of expectations on perceptual inference, for each participant we calculated the expectation-induced bias by normalizing the ratio of expectation-congruent and expectation-incongruent mean phase duration with respect to the ratio from the learning phase. Higher values indicate a stronger expectation-induced bias, hence a stronger effect of expectations on perceptual inference. Three participants were excluded from further analysis due to button malfunction.

### Statistics

Statistical analyses were conducted using the SPSS program Student version 20 and the PROCESS macro for mediation analysis (Hayes, 2013). As visual inspection of the distribution of the analyzed variables suggested non-normality, in all analyses bootstrapping methods were used to assess statistical significance (see below).

To assess the influence of genetic DA availability on the effect of expectations on perceptual inference and the tendency toward unfounded beliefs, we tested for correlations across participants between the number of COMT high-activity haplotypes (2,1 or 0) and the perceptual expectation-induced bias as well as the PDI conviction score. In order to enable ensuing mediation analysis (see below), correlation analyses were performed using linear regression models. Statistical significance of the obtained regression coefficients was assessed by obtaining 95% confidence interval by bootstrapping based on 50,000 samples.

To examine the potential mediating effect of an enhanced effect of expectations on perceptual inference on the relationship between genetic DA availability and the tendency toward unfounded beliefs, we performed a mediation analysis with expectation-induced bias as mediator variable, COMT high-activity haplotype number as independent variable and PDI conviction score as dependent variable. Statistical significance of the mediation model was tested using the bootstrapping method with bias-corrected confidence estimates as implemented in the PROCESS macro.

## Results

### COMT haplotype distribution

To address inter-individual differences in dopaminergic neurotransmission, participants were genotyped for four SNPs in the COMT gene that define previously described haplotypes with substantial impact on DA degradation, and, consequently, on synaptic DA availability (Diatchenko et al., 2005). The three most common haplotypes account for 11- to 25-fold differences in COMT enzyme activity (Diatchenko et al., 2005; Nackley et al., 2006) thereby exceeding the functional relevance of any of the individual SNPs. As expected, we found that the four examined SNPs were in strong linkage disequilibrium (all *D*′ > 0.95) confirming their location on one haplotype block. Haplotype frequencies were comparable to previous reports revealing three common haplotypes (data not shown). As an individual measure of genetically determined DA availability, we calculated the number of haplotypes with the highest enzyme function (high-activity-haplotype, also known as low-pain-sensitivity haplotype [LPS]) for each participant. This diplotype estimation revealed 14 homozygous high enzymatic activity haplotype carriers (low DA availability), 49 heterozygous high enzymatic haplotype carriers (intermediate DA availability) and 39 high-activity-haplotype non-carriers (high DA availability). Age and gender did not differ significantly between the three haplotype carrier groups (*p* > 0.1, ANOVA and chi-squared test, respectively, Table 1).

**Table 1:** **COMT genotype and demographic characteristics**.

**High-activity haplotype carrier status**	**Subjects**	**Mean age (SD)**	**Gender (M:F)**
Homozygous	14	23.8 (3.4)	5:9
Heterozygous	49	24.3 (4.2)	23:26
Non-carrier	39	26.1 (5.4)	21:18
Σ	102	24.9 (4.7)	49:53

### COMT genotype influences tendency toward unfounded beliefs

**Figure 2:**
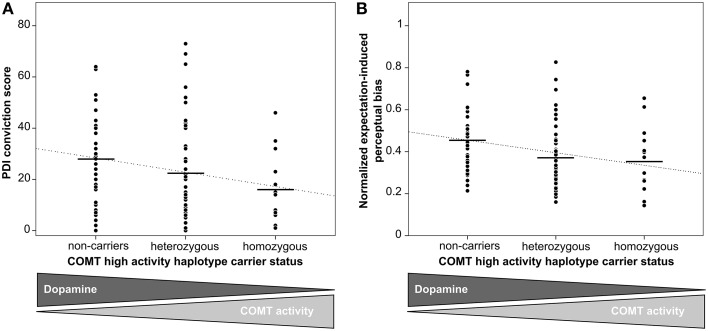
**Effect of genetically determined DA availability on unfounded beliefs and perceptual inference**. Black horizontal lines indicate genotype group means. The dotted line represents the fitted regression line. **(A)** The number of high-activity COMT haplotypes was inversely correlated to PDI conviction scores (*B* = −5.8, *SE* = 2.2, *p* = 0.01, linear regression), indicating that increasing genetic DA availability was associated with an increasing tendency toward unfounded beliefs. **(B)** The number of high-activity COMT haplotypes was inversely correlated to the expectation-induced bias on visual perception (*B* = −0.06, *SE* = 0.02, *p* = 0.01, linear regression), indicating that increasing genetic DA availability was associated with an increasing effect of expectations on perceptual inference. Expectation-induced bias was calculated by normalizing the ratio of expected and unexpected mean phase duration with respect to the ratio from the learning phase.

As excessive DA signaling has been consistently implicated in the formation of pathological unfounded beliefs, i.e., delusions (Pankow et al., 2012), we first tested whether genetic differences in dopaminergic neurotransmission are linked to inter-individual differences in the tendency toward unfounded beliefs in our sample of healthy participants. We found that the number of high-activity haplotypes was inversely correlated to the score of unfounded convictions (*B* = −5.8, *SE* = 2.2, *p* = 0.01, 95% *CI* = [−10.1−1.4], linear regression based on 50,000 bootstrapped samples, Figure 2A, see online Supplementary Material for additional analysis), indicating that higher genetically determined DA availability was associated with higher degrees of unfounded beliefs in healthy individuals.

### COMT genotype influences perceptual inference

To investigate the hypothesis that inter-individual differences in perceptual inference mechanisms might mediate the observed effect of genetic DA availability on the tendency toward unfounded beliefs, we next tested for the effect of COMT genotype on perceptual inference. Based on previous observations that perception in people with a higher propensity toward unfounded beliefs is shaped more strongly by their expectations (Tsakanikos and Reed, 2005; Krummenacher et al., 2010; Riekki and Lindeman, 2013; Van Elk, 2013), we conducted a visual perception experiment during which expectations were induced using a placebo-like manipulation (Figure 1, Sterzer et al., 2008; Schmack et al., 2013a).

As reported previously for a sample that consisted of all but one participants of our current sample as well as of three additional participants (Schmack et al., 2013a), there were significant differences between expected and unexpected percepts in the learning phase (difference of normalized percept durations 1.52 ± 0.05 [mean ± SEM], *t* = 28.43, *p* < 0.001, paired *t*-test, *p*-value based on 10,000 permutations), and, importantly also in the following test phase (0.14 ± 0.03, *t* = 4.31, *p* < 0.001, paired *t*-test, *p*-value based on 10,000 permutations), confirming that participants' expectations biased perception of the ambiguous sphere (Figure 1). The strength of the expectation-induced perceptual bias was positively correlated to the delusional conviction score, showing that in individuals with a stronger tendency toward unfounded beliefs perception was more strongly biased by expectation. Importantly, the observed effect of expectations was related to COMT genotype: we found that the number of high activity haplotypes was inversely correlated to the expectation-induced perceptual bias (*B* = −0.06, *SE* = 0.02, *p* = 0.01, 95% *CI* = [−0.10−0.02], linear regression based on 50,000 bootstrapped samples, Figure 2B, see online Supplementary Material for additional analysis). This finding suggests that higher genetically determined DA availability is related to a stronger influence of expectations on perception, thereby providing a neurobiological basis for inter-individual differences in perceptual inference.

To further elucidate the found relationship between genetic dopamine availability and perceptual inference, we tested whether the expectation-induced perceptual bias was unlearned over time. However, there was no significant difference between the expectation-induced bias in the first and in the second test run (first run 0.43 ± 0.02, second run 0.45 ± 0.03 [mean ± SEM], *p* = 0.60, *t*[101] = −0.52, paired *t*-test), rendering time effects on the strength of perceptual expectations unlikely. Furthermore, there was no relationship between high-activity haplotype number and the difference of the expectation-induced bias between the two runs (*B* = −3.2^*^10–4, 95% *CI* = [−0.070.04], linear regression based on 50,000 bootstrapped samples). Thus, our current results do not provide any evidence that the stronger effect of expectations on perception in individuals with higher genetically determined DA availability was due to weaker unlearning of expectations over time.

### Perceptual inference mediates effect of COMT haplotypes on tendency toward unfounded beliefs

**Figure 3:**
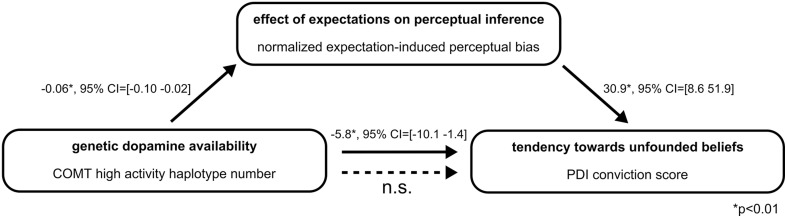
**Summary of the mediation analysis**. The association of genetic dopamine availability and tendency toward unfounded beliefs is significantly reduced to non-significant values when the measure of the effect of expectations on perceptual inference is included in the model (indirect effect *B* = −1.5, 95% *CI* = [−4.2−0.2], mediation analysis based on 50,000 bootstrapped samples, Hayes, 2013).

To formally test our hypothesis that inter-individual differences in perceptual inference might mediate the observed connection between genetic DA availability and the tendency toward unfounded beliefs, in a final step we performed a statistical mediation analysis. The mediation model included COMT high activity haplotype number as independent variable, delusional conviction score as dependent variable and expectation-induced perceptual bias as mediator variable (Figure 3). As reported above, COMT high activity haplotype number was significantly associated with delusional conviction scores (*B* = −5.8, *SE* = 2.2, *p* = 0.01, 95% *CI* = [−10.1−1.4]). Furthermore, the analysis confirmed the association between COMT high activity haplotype number and the mediator expectation-induced perceptual bias (*B* = −0.06, *SE* = 0.02, *p* = 0.01, 95% *CI* = [−0.10−0.02]). As reported in a previous publication from the same sample (Schmack et al., 2013a), there was also a significant association between the mediator expectation-induced perceptual bias and delusional conviction scores (*B* = 30.9, *SE* = 11.0, *p* < 0.01, 95% *CI* = [8.651.9]). Notably, when the measure of expectation-induced perceptual bias was included in the model, the association between COMT high activity haplotype number and delusional conviction scores was reduced to non-significant values (*B* = −4.2, *SE* = 2.4, *p* > 0.05, 95% *CI* = [−8.90.5] including zero), and this reduction was significant (indirect effect *B* = −1.5, *SE* = 1.0, 95% *CI* = [−4.2−0.2], mediation analysis based on 50,000 bootstrapped samples, Hayes, 2013). Thus, as summarized in Figure 3, the association between genetic DA availability and tendency toward unfounded beliefs was fully mediated by the measure expectation-induced perceptual bias. Although, care should be taken when drawing conclusions about causality, these results provide supportive evidence for our hypothesis that inter-individual differences in perceptual inference might provide the link between genetically determined DA availability and the tendency toward unfounded beliefs.

## Discussion

Our current study, to the best of our knowledge, is the first to demonstrate that genetic differences in DA neurotransmission are associated with inter-individual differences in perceptual inference that are linked to the formation and maintenance of unfounded beliefs involving suspicious thoughts, grandiosity ideas or beliefs into the paranormal. We found that increased DA availability, as determined by a decreasing number of COMT high-activity haplotypes, is associated with, first, a stronger tendency toward unfounded beliefs, and, second, an increasing effect of expectations on visual perception. In addition, we show that the effect of genetically determined DA availability on perceptual inference statistically mediates the effect of genetically determined DA availability on unfounded beliefs. Taken together, our results suggest that DA plays a central role in the mechanisms that govern perceptual inference, which might provide a neurobiological basis for common unfounded beliefs.

DA has been implicated in a vast variety of cognitive, motivational and motor functions (Schultz, 2007) and in the expression of consciousness (Palmiter, 2011). Although, it has been conceptualized to play an essential role in encoding precision in prediction error signaling and thereby balancing the influence of top-down expectations with bottom-up sensory information during active inference (Friston et al., 2014, 2012), evidence regarding its role in perceptual inference has remained sparse (Schmack et al., 2013b). This is even more surprising given the fact that an excess of DA has been consistently linked to the formation of pathological delusional beliefs (Pankow et al., 2012) that in turn can be explained by a dysfunction in the mechanisms underlying perceptual inference and learning. In this context, it has been proposed that exaggerated dopaminergic neurotransmission corresponds to an increased precision in prediction error signaling (Adams et al., 2013). Such prediction error signals are assumed to drive in a feedforward manner maladaptive learning processes that lead to the formation of unfounded beliefs (Kapur, 2003; Fletcher and Frith, 2009; Heinz and Schlagenhauf, 2010) and may result in enhanced feedback signals on sensory processing that account for the maintenance of unfounded beliefs (Corlett et al., 2009; Schmack et al., 2013a). However, empirical evidence corroborating the hypothesized link between DA and perceptual inference has remained sparse and inconclusive. A recent fMRI study linked activations in dopaminergic midbrain areas to sensory prediction errors during visual learning (Iglesias et al., 2013), indirectly supporting the involvement of DA in perceptual inference. Interestingly, a placebo-controlled study with L-DOPA showed that enhanced DA neurotransmission increases the tendency to perceive expected yet non-existent contents in visual noise (Krummenacher et al., 2010). This effect was observed only in individuals with low degrees of unfounded beliefs, whereas in individuals with high degrees it was reversed, suggesting complex dopamine effects on perceptual inference. Here, we demonstrate a linear relationship between synaptic DA availability and the effect of expectations on perceptual inference. Thus, together with previous work, we provide evidence that DA might be critically involved in feedback signals on sensory processing that align perception with expectations and beliefs and thus facilitate the awareness of the environment and self. We suggest that enhanced dopaminergic neurotransmission might bias perceptual inference toward a state that facilitates the persistence of unfounded beliefs.

It is tempting to speculate that DA signaling in the PFC might mediate the observed effect of COMT haplotype on perceptual inference, and thus, unfounded beliefs (Egan et al., 2001; Joober et al., 2002; Malhotra et al., 2002). Interestingly, and in line with our findings, a recent study on the *val*^158^*met (rs4680)* polymorphism in the COMT gene showed that differences in DA availability are associated to differences in the susceptibility to interpret incoming information in accordance with learned prior beliefs (Doll et al., 2011). COMT substantially modulates prefrontal DA. In rodents, both pharmacological inhibition and genetic knockout of COMT enhanced prefrontal dopaminergic neurotransmission (Tunbridge et al., 2004; Yavich et al., 2007; Lapish et al., 2009; Käenmäki et al., 2010). In humans, genetic differences in COMT function are associated with differences in performance and evoked neural activity during cognitive tasks that depend on prefrontal DA. Thus, COMT haplotypes with impact on DA metabolism might directly influence prefrontal DA signaling. On the other hand, dopaminergic neurotransmission in the PFC seems to be critically involved in perceptual inference. In rodents, feedback signals from the PFC influence sensory processing in the visual cortex (Tomita et al., 1999; Moore and Armstrong, 2003). Moreover, functional neuroimaging studies in humans have consistently reported that attention and expectation during perceptual inference not only alter activity in visual cortex, but also involve PFC activity (Kastner et al., 1999; Bar and Kassam, 2006; Summerfield and Koechlin, 2008), consistent with the proposed role of the PFC in modulating sensory processing via feedback signals. Most importantly, in a previous fMRI study we applied the same task as in the current experiment to an independent cohort of participants, and found that expectations biased sensory processing in visual cortex and were associated with enhanced neural responses in orbital PFC. Moreover, functional connectivity between orbital PFC and motion-sensitive visual cortex was related to the tendency toward unfounded beliefs (Schmack et al., 2013a). These findings suggest that feedback signals from the PFC to the visual cortex might shape perceptual inference into conformity with beliefs, thereby accounting for the endorsement of unfounded beliefs. In conclusion, there is convincing evidence for a substantial impact of COMT on dopaminergic neurotransmission in the PFC as well as for a crucial role of the PFC in perceptual inference. Thus, we propose that haplotype-associated differences in COMT function translate to individual differences in prefrontal DA availability, which in turn influence feedback signals to sensory areas involved in the molding effect of belief-driven expectations on perceptual inference and, thus, in the maintenance of unfounded beliefs.

However, a direct link between enhanced prefrontal DA signaling and a stronger tendency toward unfounded beliefs remains to be demonstrated. Conditions associated with pathological forms of unfounded beliefs (i.e., delusions) such as schizophrenia have been proposed to correspond to a decrease rather than an increase in prefrontal DA signaling (Davis et al., 1991), but these accounts have not referred to unfounded beliefs but other symptoms of schizophrenia. Furthermore, although neuroimaging studies in humans that have associated delusions with altered PFC activation during incentive and associative learning (Corlett et al., 2007; Schlagenhauf et al., 2009), conclusive evidence on the role of prefrontal DA signaling in pathological forms of unfounded beliefs is still lacking (Howes and Kapur, 2009). This is in contrast to the well-established association of excessive subcortical DA signaling with delusions (Howes and Kapur, 2009). Therefore, it is also conceivable that subcortical DA signaling might be involved in the observed effect of COMT haplotypes on perceptual inference, and thus, unfounded beliefs. Recent neurochemical work in rodents has indeed indicated an influence of COMT on striatal DA neurotransmission (Laatikainen et al., 2013), and functional neuroimaging studies in humans have shown that genetic differences in COMT function affect striatal responses during reward processing, for which DA is crucial (Yacubian et al., 2007; Schmack et al., 2008). In this context it is noteworthy that a recent functional neuroimaging study in humans has indicated that dopaminergic midbrain might encode the precision during cognitive inference, suggesting a generic role for subcortical DA in inference (Schwartenbeck et al., 2014). Thus, COMT haplotypes might directly affect DA signaling in subcortical dopaminergic regions, which might, most likely mediated by cortical regions including PFC, influence perceptual inference, thereby accounting for unfounded beliefs. Future work is needed to investigate the exact mechanisms underlying our current results.

Unfounded beliefs in the general population have been suggested to lie on a dimensional continuum with delusions in psychotic patients suffering from schizophrenia (Freeman, 2006; Linscott and van Os, 2013) that in turn are related to excessive DA signaling (Howes and Kapur, 2009; Pankow et al., 2012). Our finding of an association between the propensity toward unfounded beliefs and genetic DA availability supports this continuity view of psychosis. Previous evidence on the relationship between schizotypal traits that are thought to represent subclinical expressions of schizophrenia in the general population and genetic variation in dopaminergic neurotransmission have remained inconclusive (Mohr and Ettinger, 2014). In this context, the most frequently studied genetic variation has been the functionally relevant *val*^158^*met (rs4680)* polymorphism in the COMT gene. The *val*^158^ allele is associated with 3 to 4 times higher enzyme function than the *met*^158^ allele, and hence lower DA levels. While some studies failed to show an association between schizotypal traits and the *val*^158^*met* polymorphism (Ma et al., 2007; Raz et al., 2008; Savitz et al., 2010), others reported this association but results were mixed with regard to the direction of the association and the affected schizotypy subdimensions (Avramopoulos and Stefanis, 2002; Stefanis and Os, 2004; Smyrnis et al., 2007; Sheldrick et al., 2008; Grant et al., 2013). One potential reason for these inconsistencies might be the heterogeneity of the symptoms that are subsumed under the construct of schizotypy (Mohr and Ettinger, 2014). Moreover, the functional relevance of the *val*^158^*met* polymorphism critically depends on other polymorphisms in the COMT gene (Diatchenko et al., 2005; Nackley et al., 2006). Here, we focused on the propensity toward unfounded beliefs, and found an association of this single trait to common haplotypes that exceed the functional relevance of the *val*^158^*met* polymorphism by accounting for 11- to 25-fold differences in COMT activity, and hence DA levels. Interestingly, the high-activity *val*^158^ allele defines both the high-activity and the low-activity haplotype, depending on the alleles of other polymorphisms. Thus, although our current results await replication, they suggest that COMT haplotypes might be a powerful surrogate marker of genetic DA availability for the study of unfounded beliefs.

In conclusion, our current findings demonstrate that genetic differences in dopaminergic neurotransmission account for inter-individual differences in perception related to inter-individual differences in beliefs. Our study therefore substantially contributes to a neurobiopsychological understanding of the variability in individual attitudes of the self toward the world, ranging from critical skepticism through unfounded convictions to distressing delusions.

## Author contributions

KS, PS, and PP conceived and designed research. KS, HR, and MS performed research. EB and DM contributed new reagents/analytic tools. KS and HR analyzed data supervised by PS. KS, HR, MS, EB, DM, PP, and PS discussed the results. KS and HR wrote the paper. KS, HR, MS, EB, DM, PP and PS gave final approval of the version to be published.

### Conflict of interest statement

The authors declare that the research was conducted in the absence of any commercial or financial relationships that could be construed as a potential conflict of interest.
