# Supplementary material for: Linking unfounded beliefs to genetic dopamine availability
Source: Front Hum Neurosci. 2015 Sep 30;9:521. doi: 10.3389/fnhum.2015.00521 (PMC4588007; doi:10.3389/fnhum.2015.00521)
Supplement: Supplementary file 1 [file DataSheet1.DOC]

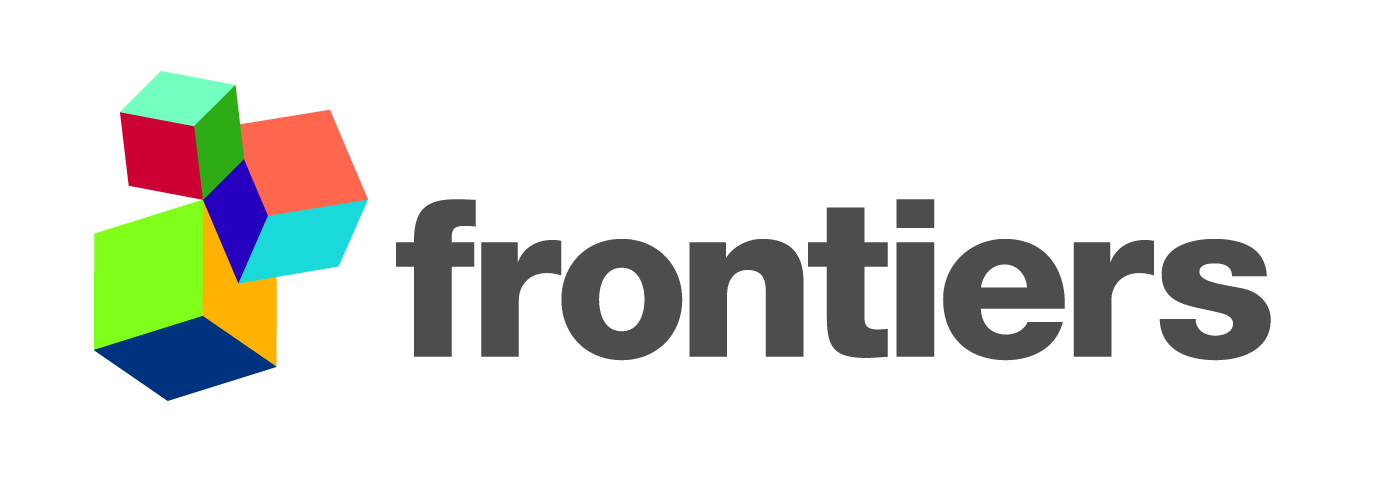
Supplementary Material

# Linking unfounded beliefs to genetic dopamine availability

**Katharina Schmack*, Hannes Rössler, Maria Sekutowicz, Eva J. Brandl, Daniel J. Müller, Predrag Petrovic, Philipp Sterzer**

*** Correspondence:** Katharina Schmack: katharina.schmack@charite.de

# Supplementary Data

**SNP-effects on expectation-induced perceptual bias and on PDI conviction score.** In order to investigate the contributions of each SNP analysed in the present study to the reported effects of genetic dopamine availability on perceptual inference and unfounded beliefs, we conducted a set of additional linear regression analyses in analogy to our main analyses. The independent variables of these analyses were the allele numbers of each of the four SNPs, the dependent variables were either the expectation-induced perceptual bias or the PDI conviction scores, yielding a total of eight analyses. Statistical significance was assessed by bootstrapping with 50,000 samples. These analyses revealed that all of the SNPs correlated significantly with the normalised expectation-induced perceptual bias (*val^158^met or rs4680 - number of val-alleles*: B=-0.048, SE= 0.02 , p=0.023, 95% CI=[-0.087 -0.009]; *rs4818 - number of G-alleles*: B=-0.06, SE=0.022, p=0.007, 95% CI=[-0.103 -0.017]; *rs4633 - number of T-alleles*: B=0.048, SE=0.02, p=0.022, 95% CI=[0.010 0.087]; *rs6269 - number of G-alleles*: B=-0.06, SE=0.022, p=0.007, 95% CI=[-0.103 -0.017], linear regression analyses based on 50,000 bootstrapped samples), whereas only two of the SNPs correlated significantly with the PDI conviction score (*rs4818 - number of G-alleles*: B=-5.823, SE=2.189, p=0.023, 95% CI=[-10.074 -1.478]; *rs6269 - number of G-alleles*: B=-5.823, SE=2.217, p=0.023, 95% CI=[-10.141 -1.437], linear regression analyses based on 50,000 bootstrapped samples). Two SNPs, including *val^158^met (rs4680)*, however, did not show a significant correlation with the PDI conviction score (*rs4680 - number of val-alleles*: B=-3.953, SE=2.141, p=0.105, 95% CI=[-8.136 0.301] including zero; *rs4633 - number of T-alleles*: B=3.969, SE=2.135, p=0.103 95% CI=[-0.261 8.114] including zero, linear regression analyses based on 50,000 bootstrapped samples).
